# Presence and Levels of Galactosyllactoses and Other Oligosaccharides in Human Milk and Their Variation during Lactation and According to Maternal Phenotype

**DOI:** 10.3390/nu13072324

**Published:** 2021-07-06

**Authors:** Simone R. B. M. Eussen, Marko Mank, Robert Kottler, Xenia-Katharina Hoffmann, Alexander Behne, Erdmann Rapp, Bernd Stahl, M. Luisa Mearin, Berthold Koletzko

**Affiliations:** 1 Danone Nutricia Research, 3584 CT Utrecht, The Netherlands; marko.mank@danone.com (M.M.); bernd.stahl@danone.com (B.S.); 2 glyXera GmbH, 39120 Magdeburg, Germany; r.kottler@glyxera.com (R.K.); x.hoffmann@glyxera.com (X.-K.H.); a.behne@glyxera.com (A.B.); e.rapp@glyxera.com (E.R.); 3 Max Planck Institute for Dynamics of Complex Technical Systems, 39106 Magdeburg, Germany; 4 Department of Chemical Biology & Drug Discovery, Utrecht Institute for Pharmaceutical Sciences, Utrecht University, 3584 CG Utrecht, The Netherlands; 5 Department of Pediatrics, Leiden University Medical Center, 2333 ZA Leiden, The Netherlands; m.l.mearin_manrique@lumc.nl; 6 Department of Paediatrics, Ludwig-Maximilians-Universität Munich, Dr. von Hauner Children’s Hospital, University of Munich Hospitals, 80337 Munich, Germany; berthold.koletzko@med.uni-muenchen.de

**Keywords:** human milk, oligosaccharides, HMOS, galactosyllactoses, lactation, human milk groups

## Abstract

Among the human milk oligosaccharides (HMOS), the galactosyllactoses (GLs) are only limitedly studied. This study aims to describe the presence and relative levels of HMOS, including GLs, in human milk (HM) according to maternal Secretor and Lewis (*SeLe*) phenotype and lactation stage. Relative levels of 19 HMOS were measured in 715 HM samples collected in the first 4 months postpartum from 371 donors participating in the PreventCD study. From a subset of 24 Dutch women (171 HM samples), samples were collected monthly up to 12 months postpartum and were additionally analyzed for relative and absolute levels of β6′-GL, β3′-GL and α3′-GL. Maternal *SeLe* phenotype or HM group was assigned based on the presence of specific fucosylated HMOS. Most HMOS, including β6′- and β3′-GL, were present in the vast majority (≥75%) of HM samples, whereas others (e.g., LNDFH II, 2′-F-LNH and α3′-GL) only occurred in a low number (<25%) of samples. Clear differences were observed between the presence and relative levels of the HMOS according to the maternal phenotype and lactation stage. Absolute concentrations of β6′-GL and β3′-GL were higher in HM group IV samples compared to samples of the other three HM groups. β3′-GL was also higher in HM group II samples compared to HM group I samples. β3′-GL and β6′-GL were stable over lactation stages. In conclusion, presence and levels of HMOS vary according to HM group and lactation stage. Not all HMOS behave similarly: some HMOS depend strongly on maternal phenotype and/or lactation stage, whereas others do not. β3′-GL and β6′-GL were present in low concentrations in over 75% of the analyzed HM samples and showed differences between HM groups, but not between the lactation stages.

## 1. Introduction

Human milk oligosaccharides (HMOS) are the third most abundant solid component in human milk (HM) after lactose and lipids, reaching between 5 and 20 g/L in mature HM [1]. The concentrations of oligosaccharides in HM vary within and among women and are influenced by various factors, including the stage of lactation, parity, maternal diet, body mass index, ethnicity, socioeconomic status and genetic predisposition [2,3,4,5,6,7,8,9,10]. Maternal genetic predisposition, particularly the individual expression pattern of Secretor *(Se)* and Lewis *(Le)* blood group genes [2,11,12,13,14], has a huge influence on the oligosaccharides profile of HM. The α1-2-fucosyltransferase gene (FUT2) is responsible for producing the *Se*-enzyme, which is necessary for generating α1-2 fucosylated HMOS, whereas the *Le* α1-3/4-fucosyltransferase gene (FUT3) is responsible for encoding the enzyme fucosyltransferase III (FucT-III), which is necessary for generating α1-4 fucosylated HMOS [10]. Depending on the activity of the *Se*-gene and *Le*-gene products (i.e., *Se*-enzymes and FucT-III-enzymes), four distinct maternal phenotypes with specific and individual HMOS patterns, usually referred to as HM groups I–IV, can be characterized [4,11,15]. 

HMOS have been suggested to play an important role in healthy infants’ development, ranging from reported effects on growth [16] and early life immune function [7] to effects on the gut microbiota [17] and intestinal functions [18]. HM is proposed to comprise more than 1000 individual and unique HMOS of which only approximately 200 are fully structurally characterized [19,20,21,22,23,24]. Yet, most research in the field of breastfeeding and HM has focused on about 20 HMOS, including the most abundant ones, such as 2′-fucosyllactose (2′-FL), lacto-N-fucopentaose (LNFP) I, lacto-N-difucohexaose (LNDFH) I and lacto-N-tetraose (LNT) [1]. Recently, the galactosyllactoses (GLs), a group of small oligosaccharides, have been identified in HM [25,26]. GLs occur in the form of several structurally distinct isomers, such as β3′-galactosyllactose and β6′-galactosyllactose (β3′- and β6′-GL, respectively) which only differ in the glycosidic linkage of the terminal galactose. Although the GLs appear in low concentrations in HM, β3′-GL and β6′-GL have recently been reported to modulate major immunologic pathways of immature human intestinal cells [27] and to attenuate inflammation [25]. The anti-inflammatory effects of the GLs may be mediated via inhibition of toll-like receptor 3 (TLR3) signaling [28]. Insights into the presences of the GLs in HM are still rare [29]. Moreover, little is known about the role of maternal expression pattern of *Se* and *Le* blood group genes and lactation stage on the levels of GLs. 

This study aims to describe the presence and relative levels of HMOS in a large number of HM samples (*n* = 715) collected in the first 4 months postpartum from 371 donors participating in the PreventCD study (www.preventcd.com, accessed on 19 November 2019). In order to substantiate evidence about the presence of the low-abundant GLs in HM, relative and absolute levels of these HMOS were determined in a sample selection comprising 171 HM samples collected from 24 Dutch donors in the first year of infants’ life (Dutch subset) using an adapted analytical approach. In order to better assess the impact of maternal genetics and lactation stage on the presence and levels of specific HMOS, the results are presented separately according to maternal *SeLe* phenotype (i.e., HM group) and the stage of lactation.

## 2. Materials and Methods 

### 2.1. Human Milk Samples

The HM samples were collected from 371 mothers participating in the PreventCD study. The PreventCD study is a multicenter study aimed to assess the role of infant nutrition and also of genetic, immunologic and environmental factors on the risk of developing coeliac disease (CD) and related autoimmune phenomena [30,31] (www.preventcd.com, accessed on 19 November 2019). Participating mothers were resident in Germany, Hungary, Italy, the Netherlands and Spain. Ethical approval for the study was obtained from the Leiden University Medical Centre (LUMC) on 1 December 2006 (P06.177), as well as from the medical ethics committees of each participating center. The study complied with Good Clinical Practices (ICH-GCP) guidelines [30] and all participants signed an informed consent form. The full details of the study have been reported previously in Hogen Esch et al. [31] and Vriezinga et al. [30]. In brief, term-born infants at risk of developing CD were recruited up to the age of 3 months. Included infants had at least one first-degree family member with CD and were genotyped to be HLA-DQ2 or HLA-DQ8 positive. The infants were randomized to receive either daily 200 mg wheat gluten or placebo (200 mg/day lactose) between 4 months and 6 months of age [31]. HM samples (10–30 mL) were collected on an explorative basis. Mothers were asked to express HM manually or by pump once a month and around the same day of each month [32]. For most women (*n* = 347), samples were collected between 1 month and 4 months postpartum. For a subset comprised of 24 Dutch women, HM samples were collected monthly up to 12 months postpartum. The collected HM samples were immediately frozen and then stored overnight at −20 °C in home freezers. Afterwards, the samples were transferred to the hospital on ice, where they were defrosted, homogenized and subsequently aliquoted in 1–2 mL portions and finally stored at −80 °C until analysis. For the HMOS analysis reported here, samples were transported on dry ice to the glycoanalytical laboratory (glyXera GmbH, Magdeburg, Germany). 

### 2.2. Analysis of Human Milk Oligosaccharides

The composition of each individual HM sample with regard to its HMOS was qualitatively and quantitatively determined using highly sensitive multiplexed capillary-gel-electrophoresis with laser-induced-florescence detection (xCGE-LIF) [33] as described in detail in the Supplementary Methods.

The limit of quantification (LOQ) was determined using the signal-to-noise ratio (S/N) of each HMOS Fingerprint that was calculated according to Ullsten et al. [34]. The LOQ was defined at an S/N of 10. All HMOS-peaks ≥ LOQ were taken into account and their internal standard (IS)-normalized peak areas were calculated. For the low-abundant GLs (i.e., β6′-, β4′-, β3′- and α3′-GL), which were of particular interest in this study, the relative and absolute levels were determined down to the limit of detection (LOD), defined at an S/N of 3. Absolute concentrations (*c*) were determined according to the following formula: *c* = IS-normalized peak height of the analyte × concentration of IS × dilution factor × response factor (KIT-glyX-Quant-DPV, glyXera GmbH, Magdeburg, Germany).

### 2.3. Selection of Human Milk Oligosaccharides

A total of 200 valid peaks, synonymous with just as many HMOS, were detected in at least one of the 715 samples by xCGE-LIF. Following their abundance and/or suggested prominent role in infants’ health [1,25,27], we focused on the following subset of HMOS: 2′-FL, 3-fucosyllactose (3-FL), difucosyllactose (DFL), LNT, lacto-N-neotetraose (LNnT), LNFP I, LNFP II, LNFP III, LNFP V, LNDFH I, LNDFH II, 3′ and 6′-sialyllactose (3′- and 6′-SL), disialyllacto-N-tetraose (DSLNT), sialyllacto-N-tetraose (LST)a, LSTb and LSTc, 3′-fucosyllacto-N-hexaose (3′-F-LNH), 2′-fucosyllacto-N-hexaose (2′-F-LNH), β1-3’-galactosyllactose (β3′-GL), β1-4’-galactosyllactose (β4′-GL), β1-6’-galactosyllactose (β6′-GL) and α1-3’-galactosyllactose (α3′-GL). 

Yet, the HMOS Fingerprints demonstrated that β4′-GL was not discernible due to co-migration with 2′-FL and was, for this reason, excluded from the analysis. Additionally, co-migration was observed for LNFP I and LNFP V and, therefore, these HMOS were analyzed as the sum of both compounds, i.e., LNFP I+V. For the HM groups that contain LNFP I (i.e., HM groups I and III, Table 1), LNFP I+V is predominantly composed of LNFP I with a small contribution of LNFP V. For the HM groups that do not contain LNFP I (i.e., HM groups II and IV, Table 1), LNFP I+V is only composed of LNFP V. This selection ultimately resulted in a subset of 22 HMOS. Over all HM samples, the sum of the IS-normalized peak areas of this subset of HMOS, further referred to as ‘selected HMOS’, comprised a median (Q1–Q3) of 85.5% (82.6–87.8) of the IS-normalized total peak area of all valid HMOS peaks.

### 2.4. Maternal Secretor- and Lewis Phenotype (HM Group)

The HM samples were assigned to a maternal Secretor- and Lewis *(SeLe)* phenotype (HM group) dependent on the presence or absence of specific fucosylated HMOS (Table 1), as described before [11,33]. 

### 2.5. Data Presentation and Statistical Analysis

Data were checked for normality using the Shapiro–Wilk test and visual inspection of histograms. As data for nearly all HMOS were non-normally distributed, data are expressed as median (Q1–Q3) relative peak areas (rPA), representing the relative proportion that each HMOS contributed to the relative total peak area (rTPA) of all selected HMOS (see Section 2.3), summed up to 100%. Similarly, IS-normalized total peak area (nTPA) of selected HMOS is presented as median (Q1–Q3) and calculated as the sum of the IS-normalized selected HMOS signals (see Section 2.3). The Mann–Whitney U-test was used to assess differences in medians between the different HM groups. Bonferroni-adjusted level of statistical significance was applied to control for multiple testing. A *p*-value < 0.05 was considered statistically significant. All statistical analyses were performed with IBM^®^ SPSS^®^ Statistics version 26 (IBM Corp. Int., Armonk, NY, USA).

## 3. Results

### 3.1. Human Milk Samples 

A total of 635 HM samples were donated by 371 women in the first 4 months postpartum, whereas a total of 171 HM samples were donated by a subset of 24 Dutch women up to 12 months postpartum (Supplemental Table S1). From the original set of samples, a total of 19 HM samples derived from six donors were excluded because of incongruent HM group typing (i.e., different HM groups assignment dependent on lactation stage) and an additional three samples derived from three donors were excluded because of missing data for lactation stage. 

### 3.2. Maternal Secretor- and Lewis Phenotype (HM Group) 

A total of 99.6% of the HM samples could be categorized into one of the four described HM groups. The HM typing showed that 66.4% of the HM samples corresponded to HM group I, 20.7% to HM group II, 8.7% to HM group III and 3.8% to HM group IV. The nTPA showed that the total abundance of HMOS was highest in HM samples assigned to HM group I, closely followed by those of HM group III and II. HM samples assigned to HM group IV had about 60% of the HMOS abundance of HM group I (Table 2 and Supplemental Table S2). 

In the next sections, first the findings related to the subset of HM samples collected in the first 4 months postpartum (371 donors; in total 635 HM samples) are described, followed by the findings from the Dutch subset comprising of longitudinally collected HM samples over the full first year postpartum (24 donors; in total 171 HM samples). Relative and absolute levels of the low-abundant GLs, β6′-GL, β3′-GL and α3′-GL were only determined in the Dutch subset of HM samples. 

### 3.3. Presence of HMOS According to Maternal Secretor- and Lewis Phenotype and Lactation Stage 

Levels of 15 out of the 19 HMOS, i.e., 2′-FL, 3-FL, DFL, LNT, LNnT, LNFP I+V, LNFP II, LNFP III, 3′-SL, 6′-SL, DSLNT, LSTb, LSTc and 3′-F-LNH, were ≥LOQ in more than 75% of analyzed HM samples. Levels of LNT, LNFP I+V, LNFP III, 3′-SL, 6′-SL, DSLNT, LSTb and 3′-F-LNH were ≥LOQ in nearly all (>98%) samples (Supplemental Table S3). In contrast, some HMOS occurred only in a low number of HM samples, i.e., LNDFH II and 2′-F-LNH were ≥LOQ in only 25.2% and 12.9% of samples, respectively.

Following the assignment of the HM groups according to the presence or absence of specific fucosylated HMOS, clear differences were observed in the HMOS patterns occurring in the different HM groups (Supplemental Table S3). Levels of 2′-FL and DFL were ≥LOQ only in the vast majority of samples assigned to HM groups I and III, LNFP II level was ≥LOQ only in the vast majority of samples assigned to HM groups I and II, LNDFH I level was ≥LOQ only in the vast majority of samples assigned to HM group I and LNDFH II level was ≥LOQ only in the vast majority of samples assigned to HM group II (Supplemental Table S3). 

There were no clear trends in presence of HMOS according to the lactation stage, with the exception of LSTa which showed a significant decrease in presence in the HM samples from 1 month postpartum (≥LOQ in 86% of samples) to 4 months postpartum (≥LOQ in 37% of samples). 

HMOS presences (≥LOQ) according to HM group in the Dutch subset of HM samples collected longitudinally during the first year postpartum were very similar to the samples collected in the first 4 months postpartum (Supplemental Table S4). Interestingly, although no clear in- or decreasing trends in the presence (≥LOQ) of LSTc was found during the first 4 months, after these first months LSTc presence decreased from about 90% to 20% at month 8.

For the GLs, results show that β6′-GL, β3′-GL and α3′-GL were detected (≥LOD) in 100%, 82% and only 1% of HM samples, respectively. β3′-GL was detected in nearly all (≥95%) samples of HM groups II and IV, compared to presences in 83% and 73% of samples assigned to HM group I and III, respectively (Supplemental Table S4). 

### 3.4. Relative Levels of HMOS According to Maternal Secretor- and Lewis Phenotype and Lactation Stage 

**Figure 1:**
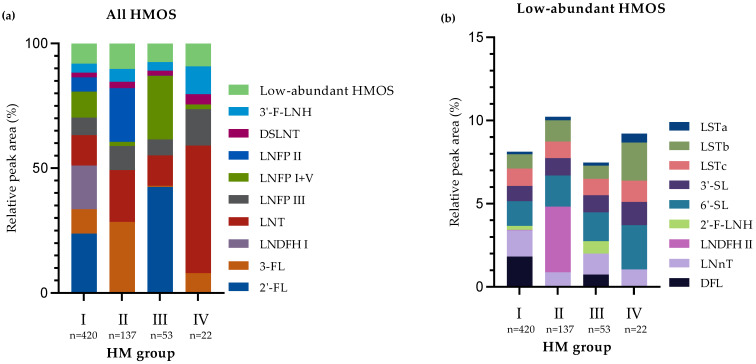
Median relative peak areas (rPA) of all selected human milk oligosaccharides (HMOS) (**a**) and of the subset of low-abundant HMOS (**b**) in the human milk (HM) samples (*n* = 635) collected in the first 4 months of lactation according to the HM groups [4,11,15]. Three samples could not be assigned to a HM group and are, for this reason, not depicted in these figures.

Figure 1 and Table 2 present the median rPA of the selected HMOS in the 635 HM samples collected in the first 4 months postpartum according to HM group. As expected, clear differences in HMOS were observed between the four known different HM groups [4,11,15]. HM group I is particularly high in 2′-FL (median rPA: 23.2%) and LNDFH I (17.3%); HM group II is particularly high in 3-FL (28.4%), LNFP II (20.6%) and LNT (19.1%); HM group III is particularly high in 2′-FL (40.9%) and LNFP I+V (24.9%); and HM group IV is particularly high in LNT (51.8%) and LNFP III (14.3%) (Figure 1 and Table 2). 

The nTPA showed that the total levels of the HMOS decreased to less than half during the first 4 months of lactation. 

**Figure 2:**
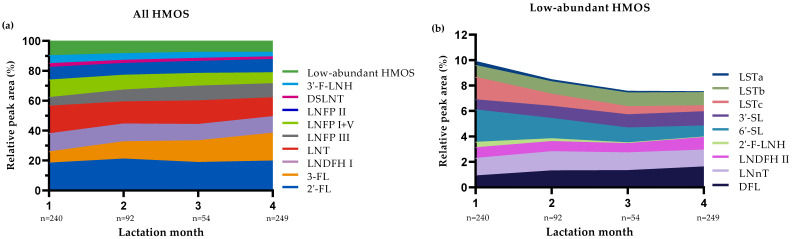
Median relative peak areas (rPA) of all selected human milk oligosaccharides (HMOS) (**a**) and of the subset of low-abundant HMOS (**b**) in the human milk samples (*n* = 635) according to lactation stage.

**Figure 3:**
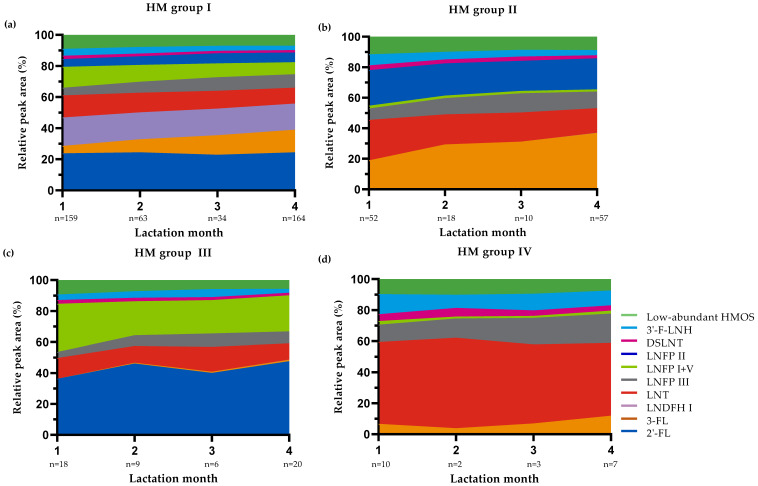
Median relative peak areas (rPA) of all selected human milk oligosaccharides (HMOS) in the human milk (HM) samples (*n* = 635) collected in the first 4 months of lactation according to HM group [4,11,15] and lactation stage. (**a**) HM group I; (**b**): HM group II; (**c**): HM group III and (**d**): HM group IV. Three samples could not be assigned to a HM group and are for this reason not depicted in these figures.

Figure 2 presents the median rPA of the selected HMOS in the 635 HM samples collected in the first 4 months postpartum according to lactation stage. The results show that the rPA of 3-FL, DFL, LNFP III and 3′-SL increased over lactation, whereas the rPA of LNT, LNFP I + V, 6′-SL, DSLNT, LSTa, LSTc, 3′-F-LNH and 2′-F-LNH decreased. Other HMOS such as 2′-FL, LNnT, LNFP II, LNDFH I, LNDFH II and LSTb were stable (i.e., no consistent in- or decreasing trends and/or <10% in- or decrease compared to previous lactation month(s)) during the first 4 months of lactation. Similar findings were observed when samples were distinguished according to HM group (Figure 3).

Median rPA of the selected HMOS according to HM group in the Dutch subset of HM samples collected during the first year postpartum were very similar to the samples collected in the first 4 months of lactation. 

β3′-GL and β6′-GL appeared in low rPA, with significantly higher levels in samples assigned to HM group IV (β3′-GL (median rPA): 0.42%; β6′-GL: 1.07%) compared to HM group I (β3′-GL: 0.074%; β6′-GL: 0.36%), group II (β3′-GL: 0.10%; β6′-GL: 0.52%) and group III (β3′-GL: 0.11%; β6′-GL: 0.42%). rPA of β6′-GL was also significantly higher in samples assigned to HM group II compared to HM group I (Supplemental Table S2). 

Only 2 out of 171 HM samples (1.2%) derived from one donor at lactation month 8 and 9 showed levels of α3′-GL ≥ LOD.

**Figure 4:**
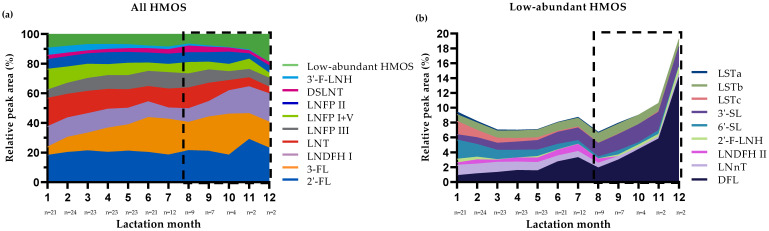
Median relative peak areas (rPA) of all selected human milk oligosaccharides (HMOS) (**a**) and of the subset of low-abundant HMOS (**b**) in the human milk (HM) samples (*n* = 171, Dutch subset) collected in the first year postpartum according to lactation month. Symbols used: 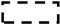, results from postpartum months 8 to 12 are based on samples from <10 participants and should be interpreted with caution.

Figure 4 presents the median rPA of the selected HMOS in the HM samples per month during the first year postpartum. 

The increasing trends in rPA observed for 3-FL, DFL, LNFP III and 3′-SL as well as the decreasing trends observed for LNT, LNFP I+V, 6′-SL, LSTa, LSTc, 3′-F-LNH and 2′-F-LNH in the first 4 months of lactation continued in subsequent postpartum months (Figure 4). In contrast, after the decline in rPA observed in the first 4 months, DSLNT slightly increased after this period. rPA of other HMOS, including β3’-GL and β6’-GL, remained stable over lactation. 

The nTPA showed that, following the initial decrease in the total amount of selected HMOS to about half in the first 4 months of lactation, HMOS amounts remained largely stable until the end of sample collection (month 12). It should be noted that the results from postpartum months 8 to 12 are based on samples from <10 participants.

### 3.5. Absolute Concentrations of GLs according to Maternal Secretor- and Lewis Phenotype and Lactation Stage

Median absolute concentrations of the GLs, analyzed in the subset of 171 HM samples, were 2.42 mg/L and 8.04 mg/L for β3′-GL and β6′-GL, respectively. 

**Figure 5:**
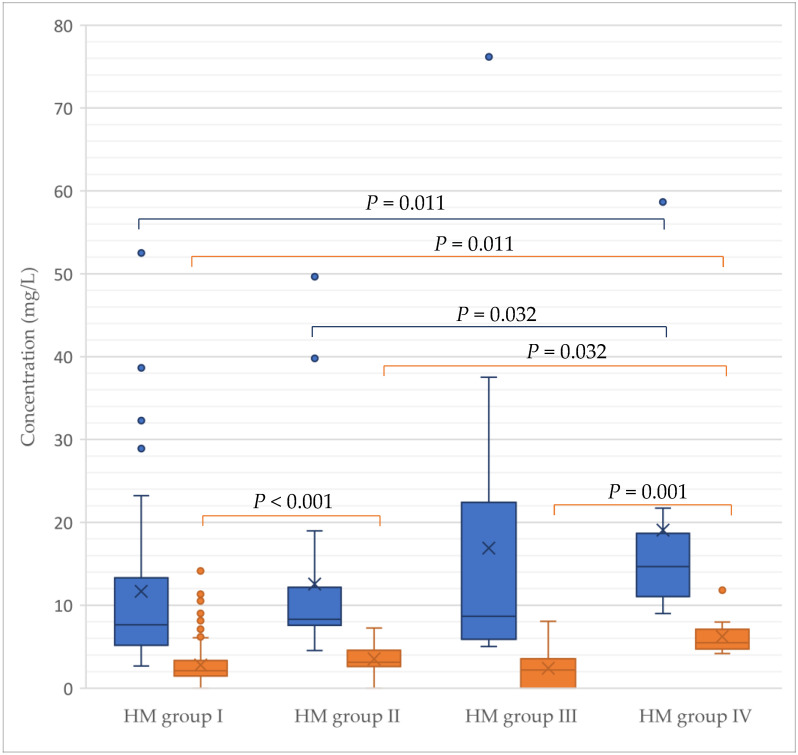
Absolute concentrations of β1-6’-galactosyllactose (β6′-GL) and β1-3’-galactosyllactose (β3′-GL) in the human milk (HM) samples in the first year of lactation according to HM group [4,11,15]. One sample assigned to HM group I with a β6′-GL concentration of 210.1 mg/L was not included in the figure for display purposes but was included in the statistical analysis. Significance values have been adjusted by the Bonferroni correction for multiple tests. A *p*-value < 0.05 was considered statistically significant.

Absolute concentrations of β3′-GL were higher in HM samples assigned to HM group IV (5.48 mg/L) compared to samples assigned to HM group I (2.12 mg/L), HM group II (3.16 mg/L) and HM group III (2.21 mg/L) (*p* < 0.05 for all). Additionally, samples of HM group II had higher absolute concentrations of β3′-GL compared to samples of HM group I (*p* < 0.001) (Figure 5). 

**Figure 6:**
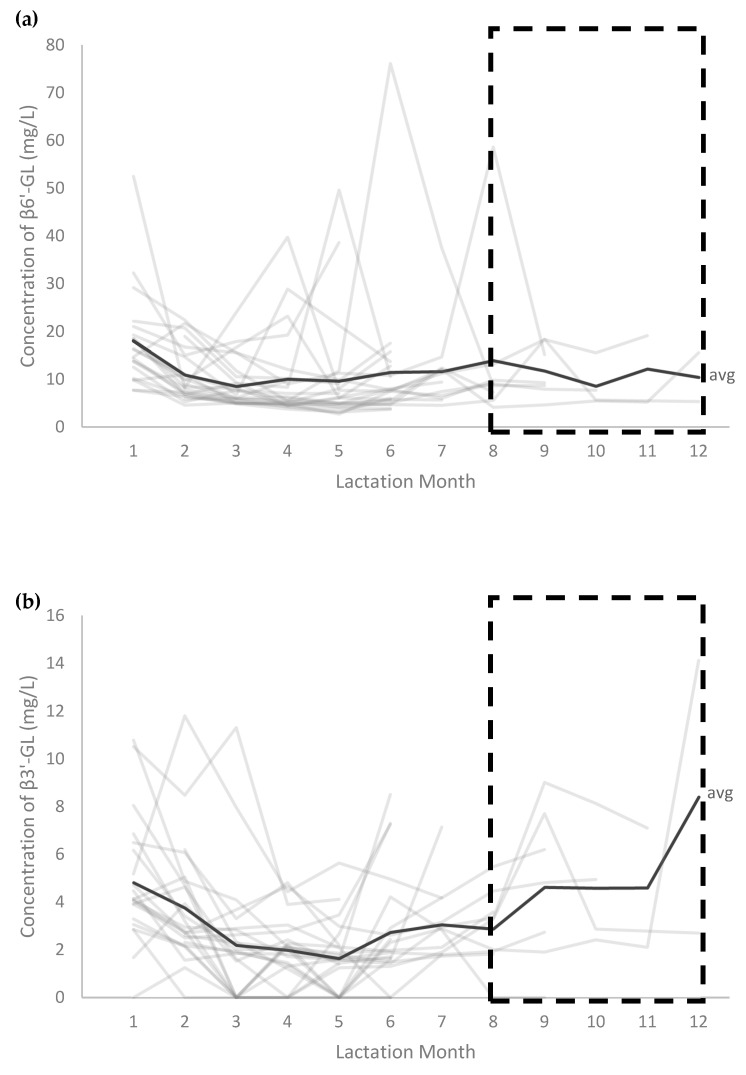
Absolute concentrations of β1-6’-galactosyllactose (β6′-GL) (**a**) and β1-3’-Galactosyllactose (β3′-GL) (**b**) in the human milk (HM) samples throughout the first year of lactation according to lactation month. One HM group I sample collected at lactation month 5 with a determined β6′-GL concentration of 210.1 mg/L was not included in the figure for display purposes. Abbreviation/symbols used: avg, average; 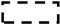, results from postpartum months 8 to 12 are based on samples from <10 participants and should be interpreted with caution.

The absolute concentration of β6′-GL was also higher in samples assigned to HM group IV (14.7 mg/L) compared to HM groups I (7.62 mg/L) and II (8.30 mg/L) (*p* < 0.05 for all) (Figure 5). Absolute levels of β3’-GL and β6’-GL remained stable over lactation stages (Figure 6). 

Only 2 out of 171 HM samples (1.2%) derived from one donor at month 8 and 9 showed absolute levels of α3′-GL ≥ LOD. The α3′-GL concentrations in those samples were 3.35 mg/L and 7.74 mg/L, respectively.

## 4. Discussion

The World Health Organization (WHO) recommends HM as the sole source of nutrition for infants during their first 6 months of life and continued breastfeeding up to two years and beyond [36]. The composition of HM adapts to the changing needs of the infant to provide the newborn with appropriate nutrients and bioactive components for each stage of growth [37]. HMOS are one of the key components of HM contributing to the healthy growth and development of the infant, including its immune system and gut microbiota [7,16,17].

In this work, we report the presence and relative levels of HMOS in a large set of HM samples (*n* = 715) collected from 371 mothers with a term-born infant at risk of developing CD residing in Germany, Hungary, Italy, the Netherlands or Spain over the first 4 months postpartum and a subset of 24 Dutch women over the first year postpartum (Supplemental Table S1). To the best of our knowledge, our study is one of the largest studies to date describing relative levels of a high number of HMOS and collecting HM samples on a monthly basis. Our results show that the composition of HM with regards to HMOS is highly variable according to maternal *SeLe* phenotype, i.e., the four HM groups. Yet not all HMOS behave similarly. 2′-FL, DFL, LNFP I, LNFP II and LNDFH I and II are examples of HMOS for which their presence in HM is heavily dependent on maternal genetics, whereas the sialyllactoses (3′- and 6′-SL) and the sialyl-lacto-N-tetraoses (LSTa, LSTb and LSTc) are very similar across the different HM groups. This finding is in line with previous studies [2,3,4,10,15]. 

A total of 99.6% of the HM samples could be categorized into one of the four HM groups. Most HM samples were categorized as HM group I (66.4%), while 20.7% were assigned to HM group II, 8.7% to HM group III and 3.8% to HM group IV. This is nicely in line with the distribution of *Se* and *Le* polymorphisms as reported by Oriol et al. (HM group I: 69%; HM group II: 20%; HM group III: 9% and HM group IV: 1%) [14]. Three HM samples (0.4% of all samples) could not be grouped into one of the four HM groups. These HM samples had in common the absence or very low relative levels of 2′-FL, DFL and LNFP I, whereas—in contrast to expectations—LNDFH I was abundantly present. LNDFH I is an α1-2-fucosylated HMOS and its presence indicates a functional FUT2 allele. The finding that other α1-2-fucosylated HMOS such as 2′-FL, DFL and LNFP I were absent despite FUT2 activity is unusual, but yet in agreement with a recent study of Durham et al. [38]. This study describes two subjects for which the maternal secretor status (determined through FUT2 genotyping based on the single-nucleotide polymorphism at r2516246) and HMOS phenotype were not in alignment, i.e., these mothers had no or low levels of 2′-FL and LNFP I despite being genotyped as secretor [38]. 

A plausible explanation of this phenomenon could be the presence and activity of another α1-2-fucosyltransferase different from the well know *Se*-enzyme. The *Se*-enzyme may catalyze synthesis of 2′-FL from lactose, DFL from 3′-FL and LNFP I from LNT [29]. A hypothetic additional novel α1-2-fucosyltransferase which could be the product of a currently unknown FUT2 polymorphism may catalyze the synthesis of LNDFH I from LNFP II. This synthesis may occur in competition with or independent from the *Se*-enzyme. Hence, even if the *Se*-enzyme is inactive or only expressed in low levels, the novel α1-2-fucosyltransferase might still catalyze formation of LNDFH I from LNFP II, whereas 2′-FL, DFL and LNFP I might not be present or only found in low levels. As the detection of LNDFH I in the discussed three HM samples still indicates presence of α1-2-fucosylated HMOS (but in an unusual pattern compared to regular HM group I specimen), but on the other hand also FucT-III-enzyme dependent HMOS (e.g., 3-FL, LNFP II and LNDFH II) are found, this finding could indicate the existence of a possible subgroup of HM group I. This new hypothetical HM group might be different from the hypothetical HM group Ia as recently postulated by Mank et al. [29] and therefore could be named HM group Ib. The existence of this FUT2 polymorphism and its influence on the HMOS patterns and HM groups requires further confirmation by analyses of oligosaccharides in HM from cohorts with high numbers of subjects and may be different between geographies and ethnicities.

In contrast to earlier findings showing that LNDFH II is present in both HM groups I and II [10], we found that only 2.1% of the analyzed HM samples in group I showed LNDFH II (≥LOQ) with a median rPA of 1.25% (Supplemental Table S3). So far, not many studies have reported on LNDFH II presence (≥LOD) and/or levels (≥LOQ) and therefore we cannot speculate as to whether it is a spurious result caused by a methodological reason or a true finding. Yet, the fact that in the first 4 months postpartum, 100% of the samples of HM group II contain LNDFH II advocates for the latter.

In addition to maternal genetic predisposition linked to the expression pattern of *Se* and *Le* blood group genes, total amounts of HMOS also varied according to lactation stage. In line with others (e.g., [3,4,5,39,40]), we showed that the nTPA of selected HMOS dropped sharply to about half in the first 4 months and thereafter remained largely stable until the end of the first year postpartum. Our group recently showed similar results in a large number of samples (*n* = 1203) from a birth cohort study conducted in South Germany [40]. In that study, we found that—in line with the decrease in total HMOS amounts—absolute concentrations of most individual HMOS decreased from 6 weeks to 6 months and 12 months postpartum. The added value of expressing the changes in individual HMOS as relative values is that these data are not obscured by the changes in the total amount of oligosaccharides present in HM. For example, it has been shown previously that absolute concentrations of 2′-FL and LNnT are continuously decreasing over the first months postpartum [4,16,38,40], whereas we show here that relative levels of these HMOS are very stable during this period (Figure 1, Figure 2 and Figure 3). In line with the increasing trends in absolute levels of 3-FL, 3′-SL and DFL over the first 6 to 12 months postpartum observed by Siziba et al. [40], we found increased relative levels of these HMOS (Figure 1, Figure 2 and Figure 3).

In addition to the relative quantification of the 22 HMOS, we describe also absolute concentrations of the GLs for a subset of 171 HM samples from 24 Dutch donors. The median concentrations of β3′-GL and β6′-GL were 2.42 mg/L and 8.04 mg/L, respectively, i.e., in agreement with previous findings [26]. Absolute concentrations of β6′-GL and β3′-GL were higher in HM group IV samples compared to samples of the other three HM groups. β3′-GL was also higher in HM group II samples compared to HM group I samples. Only 2 out of 171 HM samples (1.2%) derived from one donor at month 8 and 9 showed levels of α3′-GL ≥ LOD. Although the GLs have been suggested to have key roles in inflammation and immune functions, the median concentrations of the GLs are much lower than those of many other HMOS, including 2′-FL, 3-FL, LNT and LNnT and LNFP I, II and III. Yet, as for other bioactive substances, the nutritional value and bioactivity of HMOS may not depend on the dose. Indeed, studies by He et al. [27] and Newburg et al. [25] have pointed towards the involvement of GLs in major immunologic pathways and inflammation attenuation. The relevance of GLs for infant growth and development remains the subject of further research. 

Strengths of this study include the large number of HM samples collected from various centers in five countries (Germany, Hungary, Italy, the Netherlands and Spain) and the longitudinal assessment of HMOS covering the full first year postpartum. Moreover, our high-sensitive and high-throughput method of analysis (xCGE-LIF) allowed for the relative quantification of more than 200 HMOS, including the up-to-now little-studied GLs, as well as a large number of HMOS that have not yet been fully structurally characterized. 

To the best of our knowledge, our method is unique in its ability to separate over 200 different HMOS on the basis of their size, charge and shape, with only one single and simple sample preparation and one measurement. The present work focused on 22 abundant and/or prominent HMOS, comprising about 85% of the total amount of oligosaccharides in HM over all HM groups. Yet, future efforts should focus on the full characterization of the remaining HMOS. 

Despite the extremely high peak resolution and peak capacity of the xCGE-LIF-based analysis, it was still not possible to separate all HMOS. Some specific HMOS (e.g., β4′-GL, LNFP I and LNFP V in some *Se* positive donors) were not automatically discernible due to partially co-migrating or overlapping peaks. In the future, a further developed automated data analysis will enable the in silico separation of these peaks. Other limitations of our study are the low number of samples collected from 8 months postpartum and onwards resulting from the mother’s decision to stop breastfeeding. Moreover, although our study included a large number of HM samples, only a small fraction (3.8%) of samples was assigned to HM group IV due to the low frequency of this particular HM group in the general population, contributing to a lower accuracy of the data in this group. Additionally, the unequal sample sizes could have affected statistical power and Type I error rates [41]. Furthermore, for the majority of HMOS no absolute concentrations were measured, limiting the ability to directly compare our findings to previously published data. Moreover, by definition, quantitative results for the HMOS (with the exception of the GLs) were provided for HM samples with HMOS intensities at or above the LOQ. As a consequence, a (low) number of additional HM samples may contain specific HMOS (≥LOD, but <LOQ), which have not been considered because HMOS levels in these samples were <LOQ and therefore their rPA was set to 0.00. 

It should be noted that the HM in this study was collected from mothers of infants at risk of developing CD. Future research should investigate whether the presence of the HLA-DQ2 and/or HLA-DQ8 genotype in the mother or infant has an impact on HMOS abundance. Other important areas for future research include investigations of the impact of maternal diet, ethnicity and country of origin on HMOS abundance, regional variations in HMOS abundance and further exploration of the influence of HMOS on infants’ growth and development, including diseases such as CD. Moreover, potential differences in HMOS concentrations driven by maternal phenotype within the HM groups as recently described by Elwakiel et al. [3] require further investigation.

## 5. Conclusions

In conclusion, this study displays the presence and relative levels of HMOS in a large number of HM samples collected in five countries (Germany, Hungary, Italy, the Netherlands and Spain) over the first year postpartum and highlights the large intra- and inter-individual variability according to maternal *SeLe* phenotype and lactation stage. Not all HMOS behave similarly: Some HMOS were heavily dependent on maternal genetics (e.g., 2′-FL, LNFP I(+V) and LNDFH I) or lactation stage (e.g., 3-FL, 6′-SL and DFL), whereas other HMOS were very similar across the different HM groups (e.g., 3′- and 6′-SL or LST a/b/c) or lactation stages (e.g., 2′-FL, LNnT and LNDFH I). β3′-GL and β6′-GL were—in low concentrations—present in more than 75% of analyzed HM samples and showed differences between HM groups dependent on maternal genetics, but not between lactation stages. 

Further research on the impact and correlation of the presence and levels of individual HMOS on the healthy development of infants is needed.

## Figures and Tables

**Table 1:** Identification of human milk (HM) groups I to IV based on the presence or absence of specific fucosylated HMOS (adapted from Oriol et al., 1986 [14]).

HM Group	Specific Fucosylation		Prominent α1-2, α1-3 and α1-4 Fucosylated HMOS
	α1-2 *(Se)*	α1-4 *(Le)*	
**I** Lewis (a − b + c − d−) ^1^	+	+	2′-FL, 3-FL, DFL, LNFP I, LNFP II, LNFP III, LNDFH I and LNDFH II
**II** Lewis (a + b − c − d−) ^1^	−	+	3-FL, LNFP II, LNFP III and LNDFH II
**III** Lewis (a − b − c − d +) ^1^	+	−	2′-FL, 3-FL, DFL, LNFP I and LNFP III
**IV** Lewis (a − b − c + d−) ^1^	−	−	3-FL and LNFP III

^1^ According to Blank et al. [35]. α1-2 *(Se)*: The gene is responsible for encoding the *Se*-enzyme, which is necessary for generating α1-2 fucosylated HMOS; α1-4 *(Le)*: The gene is responsible for encoding the enzyme FucT-III, which is necessary for generating α1-4 fucosylated HMOS.

**Table 2:** Total peak area of the selected human milk oligosaccharides (HMOS) (nTPA) and relative peak area (rPA, %) of the HMOS (median (Q1–Q3)) according to maternal human milk (HM) group [4,11,15] in HM samples (*n* = 635) collected in the first 4 months postpartum from 371 donors. Three samples could not be assigned to a HM group and are, for this reason, not depicted in this table.

	HM Group I (*n* = 420 Samples, 241 Donors) Median (Q1–Q3)	HM Group II (*n* = 137 Samples, 83 Donors) Median (Q1–Q3)	HM Group III (*n* = 53 Samples, 32 Donors) Median (Q1–Q3)	HM Group IV (*n* = 22 Samples, 14 Donors) Median (Q1–Q3)
nTPA ^1^	12.3 (10.0–17.6) ^a^	10.7 (8.33–14.1) ^a,b^	10.9 (8.73–14.7) ^a,b^	8.18 (5.75–10.5) ^b^
**rPA (%)**				
2′-FL	23.2 (18.4–29.0) ^a^	0.00 (0.00–0.00) ^2,b^	40.9 (34.2–48.9) ^c^	0.00 (0.00–0.00) ^2,b^
3-FL	7.33 (3.70–13.8) ^a^	28.4 (17.2–35.8) ^b^	0.00 (0.00–0.00) ^2,c^	6.25 (4.03–11.3) ^a^
DFL	1.61 (1.13–2.20) ^a^	0.00 (0.00–0.00) ^2,b^	0.65 (0.33–1.08) ^c^	0.00 (0.00–0.00) ^2,b^
LNT	11.9 (9.18–14.6) ^a^	19.1 (14.5–26.5) ^b^	11.8 (9.10–13.8) ^a^	51.8 (46.4–56.7) ^c^
LNnT	1.44 (1.02–2.04) ^a^	0.68 (0.45–1.16) ^b^	1.10 (0.82–1.50) ^c^	0.91 (0.45–1.44) ^a,b,c^
LNFP I + V	9.69 (6.00–14.0) ^a^	1.65 (1.39–1.97) ^b^	24.9 (19.3–31.5) ^c^	1.60 (1.32–2.36) ^b^
LNFP II	5.13 (3.57–7.34) ^a^	20.6 (17.6–25.4) ^b^	0.00 (0.00–0.00) ^2,c^	0.00 (0.00–0.00) ^2,c^
LNFP III	6.81 (4.62–8.83) ^a^	9.25 (7.30–11.3) ^b^	6.44 (3.85–8.49) ^a^	14.3 (9.58–18.0) ^c^
LNDFH I	17.3 (14.9–20.2) ^a^	0.00 (0.00–0.00) ^2,b^	0.00 (0.00–0.00) ^2,b^	0.00 (0.00–0.00) ^2,b^
LNDFH II	0.00 (0.00–0.00) ^2,a^	3.77 (2.84–4.88) ^b^	0.00 (0.00–0.00)^2,a^	0.00 (0.00–0.00) ^2,a^
3′-SL	0.89 (0.70–1.07) ^a^	1.01 (0.82–1.25) ^b^	0.94 (0.76–1.22) ^a,b^	1.45 (1.09–1.63) ^c^
6′-SL	1.22 (0.76–2.22) ^a^	1.58 (0.92–2.67) ^a,b^	1.31 (0.88–2.51) ^a^	2.42 (1.80–3.45) ^b^
DSLNT	1.71 (1.32–2.27) ^a^	2.37 (1.69–3.13) ^b^	1.94 (1.31–2.48) ^a,b^	4.09 (3.22–5.05) ^c^
LSTa	0.17 (0.00–0.26) ^a^	0.21 (0.00–0.31) ^a^	0.00 (0.00–0.32) ^a^	0.47 (0.32–0.75) ^b^
LSTb	0.80 (0.60–1.04) ^a^	1.21 (1.03–1.49) ^b^	0.75 (0.57–0.89) ^a^	2.21 (1.89–2.75) ^c^
LSTc	0.75 (0.42–1.37) ^a^	0.75 (0.39–1.32) ^a^	0.60 (0.45–1.42) ^a^	1.01 (0.74–1.79) ^a^
3′-F-LNH	3.42 (2.35–4.59) ^a^	4.60 (2.71–7.18) ^b^	2.65 (1.99–4.72) ^a^	10.5 (8.48–11.3) ^c^
2′-F-LNH	0.00 (0.00–0.00) ^2,a^	0.00 (0.00–0.00) ^2,a^	0.00 (0.00–1.20) ^a^	0.00 (0.00–0.00) ^2,a^

^1^ Displayed as the internal standard (IS)-normalized total peak area of the selected HMOS (nTPA); ^2^ ‘0.00′ does not necessarily mean that the respective HMOS was not present at any level, but it means that any amount of HMOS present was below the limit of quantification (LOQ); ^a,b,c^ Different superscript letters indicate statistical significance (*p* < 0.05). Significance values have been adjusted by the Bonferroni correction for multiple tests. Abbreviations used: 2′-FL: 2′-fucosyllactose; 3-FL: 3-fucosyllactose; DFL: difucosyllactose; LNT: lacto-N-tetraose; LNnT: lacto-N-neotetraose; LNFP I: lacto-N-fucopentaose I; LNFP II: lacto-N-fucopentaose II; LNFP III: lacto-N-fucopentaose III; LNFP V: lacto-N-fucopentaose V; LNDFH I: lacto-N-difucohexaose I; LNDFH II: lacto-N-difucohexaose II; 3′-SL: 3-sialyllactose; 6′-SL: 6-sialyllactose; DSLNT: disialyllacto-N-tetraose; LSTa: sialyl-lacto-N-tetraose a; LSTb: sialyl-lacto-N-tetraose b; LSTc: sialyl-lacto-N-tetraose c; 2′-F-LNH: 2′- fucosyllacto-N-hexaose; 3′-F-LNH: 3′-fucosyllacto-N-hexaose.

## Data Availability

The data presented in this study are available on request from the corresponding author. A data sharing agreement will be requested.

## Supplementary Materials

The following are available online at https://www.mdpi.com/article/10.3390/nu13072324/s1, Supplementary methods, Table S1: Overview of the number of human milk (HM) samples collected at different time-points over the first 4 months (middle column) and for the subset of 24 Dutch women over the first 12 months (right column) of lactation, Table S2: Total peak area of the selected human milk oligosaccharides (HMOS) (nTPA) and relative peak area (rPA, %) of the HMOS (median (Q1–Q3)) according to maternal human milk (HM) group [4,11,15] in HM samples (*n* = 171) collected longitudinally over the first year postpartum from 24 Dutch mothers (Dutch subset), Table S3: Percentage of human milk (HM) samples (*n* = 635) in which the specific human milk oligosaccharide (HMOS) was detected (Presence, % of total) and relative peak area (rPA, %) of the selected HMOS (median (Q1–Q3)) according to maternal HM group [4,11,15]. HM samples were collected in the first 4 months postpartum from 371 donors, Table S4: Percentage of human milk (HM) samples (*n* = 171) in which the specific human milk oligosaccharide (HMOS) was detected (Presence, % of total) and relative peak area (rPA, %) of the selected HMOS (median (Q1–Q3)) according to maternal HM group [4,11,15]. HM samples were collected longitudinally over the first year postpartum from 24 Dutch mothers, Figure S1: Median relative peak areas (rPA) of low-abundant human milk oligosaccharides (HMOS) in the human milk (HM) samples (*n* = 635) collected in the first 4 months of lactation according to HM group [4,11,15] and lactation stage.

## Abbreviations

2′-FL, 2′-fucosyllactose; 2′-F-LNH, 2′-fucosyllacto-N-hexaose; 3-FL, 3-fucosyllactose; 3′-F-LNH, 3′-fucosyllacto-N-hexaose; 3′-SL, 3′-sialyllactose; 6′-SL, 6′-sialyllactose; α3′-GL, α1-3’-galactosyllactose; β3′-GL, β1-3’-galactosyllactose; β4′-GL, β1-4’-galactosyllactose; β6′-GL, β1-6’-galactosyllactose; CD, coeliac disease; DFL, difucosyllactose; DP, degree of polymerization; DSLNT, disialyllacto-N-tetraose; FucT-III, fucosyltransferase III; FUT2, α1-2-fucosyltransferase; GL, galactosyllactose; HM, human milk; HMOS, human milk oligosaccharides; IS, internal standard; *Le,* Lewis; LOD, limit of detection; LNDFH, lacto-N-difucohexaose; LNFP, lacto-N-fucopentaose; LNnT, lacto-N-neotetraose; LNT, lacto-N-tetraose; LOQ, limit of quantification; LST, sialyllacto-N-tetraose; LUMC, Leiden University Medical Centre; nTPA, IS-normalized total peak area; rPA, relative peak area; rTPA, relative total peak area; *Se,* Secretor; S/N, signal-to-noise ratio; xCGE-LIF, multiplexed capillary-gel-electrophoresis with laser-induced-florescence detection; WHO, World Health Organization.
