# Supplementary material for: Presence and Levels of Galactosyllactoses and Other Oligosaccharides in Human Milk and Their Variation during Lactation and According to Maternal Phenotype
Source: Nutrients. 2021 Jul 6;13(7):2324. doi: 10.3390/nu13072324 (PMC8308909; doi:10.3390/nu13072324)
Supplement: Supplementary file 1 [file nutrients-13-02324-s001.zip › nutrients-1232328-supplementary.pdf]

Supplementary methods

Analysis of human milk oligosaccharides

The composition of each individual HM sample with regard to its HMOS was qualitatively and quantitatively determined using highly sensitive multiplexed capillary-gel-electrophoresis with laser-induced-florescence detection (xCGE-LIF) [1] as described in the Supplementary methods.

In brief and in accordance with the glyXera GmbH kit protocol (KIT-glyX-Quant-DPV, glyXera GmbH, Magdeburg, Germany), the pure HM samples were diluted 1:100 and then spiked with an internal standard (IS) (oligosaccharide quantification standard solution, OS-A5-N-1mL-01, glyXera GmbH, Magdeburg, Germany). For this step, 2 µL of an IS solution and 3 µL of ultrapure water [2] were added to 20 µL of each diluted HM sample. Subsequently, the IS spiked samples were treated with denaturation solution (C-DeNatSol-100uL-01, glyXera GmbH, Magdeburg, Germany). Next, the free oligosaccharides within the denaturized HM/IS samples, comprising the free HMOS and IS, were labeled with APTS dye (KIT-glyX-OSP-A-96-01, glyXera GmbH, Magdeburg, Germany). Afterwards, the excess of APTS (not reacted) and salt were removed using hydrophilic interaction chromatography solid phase extraction (HILIC-SPE, in accordance with the glyXera GmbH kit protocol KIT-glyX-OS.H-APTS-96-01, glyXera GmbH, Magdeburg, Germany). In the next step, the purified APTS-labeled oligosaccharides (HILIC-SPE eluates) were analyzed via xCGE-LIF in accordance with the glyXera GmbH measurement kit protocol (KIT-glyX-OS.M-96-01, glyXera GmbH, Magdeburg, Germany). Finally, data processing, analysis, and qualitative evaluation of the HMOS *Fingerprints* (normalized electropherograms) were performed via glyXtool™ software (version 5.3.1, glyXera GmbH, Magdeburg, Germany), and data analysis and quantitative evaluation of the HMOS *Fingerprints* was performed via glyXtoolGUI™ software (Beta v0.4 [r166+], glyXera GmbH, Magdeburg, Germany).

The limit of quantification (LOQ) was determined using the signal-to-noise ratio (S/N) of each HMOS *Fingerprint* that was calculated according to Ullsten et al. [3]. The LOQ was defined at an S/N of 10. The respective noise within each sample was determined after the migration time alignment of the unsmoothed data in the late migration time range (approximation range = degree of polymerization (DP) $18<DP<20$). Consequently, the LOQ is specific with regard to each sample and measurement. All HMOS-peaks ≥LOQ were taken into account and their IS-normalized peak areas were calculated.

For the low-abundant GLs (i.e. β6′-, β4′-, β3′-, and α3′-GL), which were of particular interest in this study, the relative and absolute levels were determined down to the limit of detection (LOD), defined at an S/N of 3. Absolute concentrations (c) were determined according to the following formula: c = IS-normalized peak height of the analyte × concentration of IS × dilution factor × response factor (KIT-glyX-Quant-DPV, glyXera GmbH, Magdeburg, Germany).

Supplemental Table S1. Overview of the number of human milk (HM) samples collected at different time-points over the first 4 months (middle column) and for the subset of 24 Dutch women over the first 12 months (right column) of lactation.

**Lactation month**	**First 4 months Number of HM samples**	**First 12 months (Dutch subset) Number of HM samples**
1 month postpartum	240	21
2 months postpartum	92	24
3 months postpartum	54	23
4 months postpartum	249	23
5 months postpartum	-	23
6 months postpartum	-	21
7 months postpartum	-	12
8 months postpartum	-	9
9 months postpartum	-	7
10 months postpartum	-	4
11 months postpartum	-	2
12 months postpartum	-	2
**Total**	635 (371 donors)	171 (24 donors)

Supplemental Table S2. Total peak area of the selected human milk oligosaccharides (HMOS) (nTPA) and relative peak area (rPA, %) of the HMOS (median (Q1-Q3)) according to maternal human milk (HM) group [4–6] in HM samples (n=171) collected longitudinally over the first year postpartum from 24 Dutch mothers (Dutch subset).

	**HM Group I (*n* = 126 Samples, 18 Donors)** ***median (Q1-Q3)***	**HM Group II (*n* = 21 Samples, 3 Donors)** ***median (Q1-Q3)***	**HM Group III (*n* = 15 Samples, 2 Donors)** ***median (Q1-Q3)***	**HM Group IV (*n* = 9 Samples, 1 Donor)** ***median (Q1-Q3)***
nTPA[1]	11.4 (9.33-13.4)[a]	9.30 (7.53-10.8)[b]	9.95 (8.51-10.7)[a,b]	6.22 (5.27-9.83)[b]
		**rPA (%)**		
2′-FL	22.2 (15.0-29.5)[a]	0.00 (0.00-0.00)[2] [b]	42.0 (35.5-53.0)[c]	0.00 (0.00-0.00)[2] [b]
3-FL	11.7 (5.53-21.4)[a]	35.9 (26.4-45.3)[b]	0.00 (0.00-3.38)[c]	6.78 (3.04-7.31)[d]
DFL	1.86 (1.29-2.50)[a]	0.00 (0.00-0.00)[2] [b]	1.01 (0.62-1.48)[a]	0.00 (0.00-0.00)[2] [b]
LNT	10.6 (7.87-13.1)[a]	15.3 (11.3-20.6)[b]	9.77 (6.50-13.8)[a]	51.8 (47.8-59.3)[c]
LNnT	0.98 (0.67-1.48)[a]	0.66 (0.00-0.91)[b]	1.21 (0.83-1.78)[a]	1.35 (0.94-1.89)[a,b]
LNFP I+V	7.56 (3.29-12.5)[a]	1.51 (1.36-1.63)[2] [b]	18.8 (15.6-21.5)[c]	1.29 (1.16-1.55)[2] [b]
LNFP II	5.79 (3.31-9.01)[a]	19.7 (17.1-21.2)[b]	0.00 (0.00-0.00)[2] [c]	0.00 (0.00-0.00)[2] [c]
LNFP III	7.52 (5.66-9.12)[a]	9.28 (8.06-10.6)[b]	8.94 (6.99-12.3)[a,b]	15.6 (10.1-18.4)[b]
LNDFH I	16.3 (13.5-19.1)[a]	0.00 (0.00-0.00)[2] [b]	0.00 (0.00-0.00)[2] [b]	0.00 (0.00-0.00)[2] [b]
LNDFH II	0.00 (0.00-0.00)[2] [a]	3.50 (2.87-4.16)[b]	0.00 (0.00-0.00)[2] [a]	0.00 (0.00-0.00)[2] [a]
3′-SL	1.08 (0.78-1.43)[a]	1.37 (1.04-1.91)[b]	1.18 (1.04-2.15)[a,b]	1.76 (1.05-2.21)[c]
6′-SL	0.83 (0.47-1.64)[a]	0.70 (0.50-1.64)[a]	0.91 (0.37-1.62)[a]	1.74 (0.66-2.71)[a]
DSLNT	1.97 (1.40-2.47)[a]	3.20 (2.86-3.71)[b]	2.95 (1.94-4.10)[a,b]	5.53 (5.00-8.83)[c]
LSTa	0.00 (0.00-0.20)[a]	0.00 (0.00-0.21)[a]	0.00 (0.00-0.21)[a]	0.78 (0.75-0.93)[b]
LSTb	0.92 (0.68-1.08)[a]	1.43 (1.29-1.89)[b]	0.64 (0.51-0.98)[a]	2.36 (2.18-2.72)[c]
LSTc	0.34 (0.00-0.84)[a]	0.28 (0.00-0.77)[a]	0.26 (0.00-0.57)[a]	0.99 (0.27-1.60)[a]
3'-F-LNH	2.38 (1.42-3.85)[a]	3.65 (0.76-6.23)[ab]	2.51 (1.44-3.94)[a]	7.46 (4.94-8.41)[b]
2'-F-LNH	0.00 (0.00-0.00)[2] [a]	0.00 (0.00-0.00)[2] [a]	0.00 (0.00-0.00)[2] [a]	0.00 (0.00-0.00)[2] [a]
β3′-GL[3]	0.074 (0.049-0.12)[a]	0.10 (0.080-0.13)[a]	0.11 (0.00-0.13)[a]	0.42 (0.38-0.51)[b]
β6′-GL[3]	0.36 (0.25-0.53)[a]	0.52 (0.44-0.61)[b]	0.42 (0.38-1.01)[ab]	1.07 (0.87-1.36)[c]
α3′-GL[3]	0.00 (0.00-0.00)[2] [a]	0.00 (0.00-0.00)[2] [a]	0.00 (0.00-0.00)[2] [a]	0.00 (0.00-0.00)[2] [a]

[1] Displayed as the internal standard (IS)-normalized total peak area of selected HMOS (nTPA); [2] ‘0.00’ does not necessarily mean that the respective HMOS is not present at any level, but it means that any amount of HMOS present is below the limit of quantification (LOQ); [3] For β6′-GL, β3′-GL and α3′-GL, the limit of

detection was defined at a signal-to-noise ratio (S/N) of 3. For all other HMOS, the limit of quantification was defined at a S/N of 10; [a,b,c and d] Different superscript letters indicate statistical significance ($p<0.05$). Significance values have been adjusted by the Bonferroni correction for multiple tests.
Abbreviations used: 2′-FL: 2′-fucosyllactose; 3-FL: 3-fucosyllactose; DFL: difucosyllactose; LNT: lacto-N-tetraose; LNnT: lacto-N-neotetraose; LNFP I: lacto-N-fucopentaose I; LNFP II: lacto-N-fucopentaose II; LNFP III: lacto-N-fucopentaose III; LNFP V: lacto-N-fucopentaose V; LNDFH I: lacto-N-difucohexaose I; LNDFH II: lacto-N-difucohexaose II; 3′-SL: 3-sialyllactose; 6′-SL: 6-sialyllactose; DSLNT: disialyllacto-N-tetraose; LSTa: sialyl-lacto-N-tetraose a; LSTb: sialyl-lacto-N-tetraose b; LSTc: sialyl-lacto-N-tetraose c; 2′-F-LNH: 2′-fucosyllacto-N-hexaose; 3′-F-LNH: 3′-fucosyllacto-N-hexaose; β3′-GL: β3′-galactosyllactose; β6′-GL: β6′-galactosyllactose; α3′-GL: α3′-galactosyllactose.

Supplemental Table S3. Percentage of human milk (HM) samples (n=635) in which the specific human milk oligosaccharide (HMOS) was detected (Presence, % of total) and relative peak area (rPA, %) of the selected HMOS (median (Q1-Q3)) according to maternal HM group [4-6]. HM samples were collected in the first 4 months postpartum from 371 donors. Three samples could not be assigned to a HM group and are for this reason not depicted in this table.

	**HM Group I (*n* = 420 Samples, 241 Donors)**		**HM Group II (*n* = 137 Samples, 83 Donors)**		**HM Group III (*n* = 53 Samples, 32 Donors)**		**HM Group IV (*n* = 22 Samples, 14 Donors)**	
	**Presence *% of total*[1]**	**rPA (%) *median (Q1-Q3)***	**Presence *% of total*[1]**	**rPA (%) *median (Q1-Q3)***	**Presence *% of total*[1]**	**rPA (%) *median (Q1-Q3)***	**Presence *% of total*[1]**	**rPA (%) *median (Q1-Q3)***
2′-FL	100	23.2 (18.4-29.0)[a]	0	-	100	40.9 (34.2-48.9)[b]	0	-
3-FL	95	7.76 (4.17-14.5)[a]	100	28.4 (17.2-35.8)[b]	15	2.86 (2.47-4.18)[c]	100	6.25 (4.03-11.3)[a]
DFL	100	1.61 (1.13-2.20)[a]	4.4	0.44 (0.25-0.65)[b]	89	0.68 (0.54-1.15)[c]	4.5	1.29 (1.29-1.29)[b]
LNT	100	11.9 (9.20-14.6)[a]	99	19.3 (14.6-26.5)[b]	100	11.8 (9.10-13.8)[a]	100	51.8 (46.4-56.7)[c]
LNnT	99	1.45 (1.03-2.06)[a]	85	0.78 (0.54-1.26)[b]	98	1.10 (0.83-1.54)[c]	95	1.04 (0.45-1.50)[a,b,c]
LNFP II	100	5.14 (3.58-7.34)[a]	100	20.6 (17.6-25.4)[b]	3.8	0.18 (0.13- .)[2] [c]	0	-
LNFP III	100	6.81 (4.62-8.83)[a]	100	9.25 (7.30-11.3)[b]	100	6.44 (3.85-8.49)[a]	100	14.3 (9.58-18.0)[c]
LNFP I+V	100	9.69 (6.00-14.0)[a]	100	1.65 (1.39-1.97)[b]	100	24.9 (19.3-31.5)[c]	100	1.60 (1.32-2.36)[b]
LNDFH I	100	17.4 (14.9-20.2)[a]	1.5	1.44 (0.043- .)[2] [b]	3.8	0.59 (0.46- .)[2] [b]	0	-
LNDFH II	2.1	1.25 (0.99-1.59)[a]	100	3.77 (2.84-4.88)[b]	0	-	0	-
3′-SL	100	0.89 (0.70-1.07)[a]	100	1.00 (0.82-1.25)[b]	98	0.95 (0.77-1.22)[a,b]	100	1.45 (1.09-1.63)[c]
6′-SL	100	1.22 (0.76-2.23)[a]	100	1.58 (0.92-2.67)[a,b]	98	1.35 (0.89-2.52)[a]	100	2.42 (1.80-3.45)[b]
DSLNT	100	1.71 (1.33-2.27)[a]	99	2.37 (1.72-3.14)[b]	98	1.94 (1.33-2.48)[a,b]	100	4.09 (3.22-5.05)[c]
LSTa	60	0.24 (0.18-0.30)[a]	58	0.29 (0.23-0.44)[a]	45	0.33 (0.27-0.45)[a]	95	0.48 (0.34-0.78)[b]
LSTb	99	0.80 (0.61-1.04)[a]	99	1.21 (1.04-1.50)[b]	92	0.76 (0.62-0.93)[a]	100	2.21 (1.89-2.75)[c]
LSTc	96	0.79 (0.46-1.42)[a]	93	0.84 (0.42-1.33)[a]	92	0.71 (0.47-1.71)[a]	95	1.02 (0.76-1.82)[a]
3′-F-LNH	98	3.46 (2.40-4.61)[a]	99	4.63 (2.77-7.18)[b]	98	2.71 (2.05-4.76)[a]	100	10.5 (8.48-11.3)[c]
2′-F-LNH	12	1.72 (1.17-2.45)[a]	0	-	36	1.69 (1.04-3.19)[a]	0	-

[1] Percentage of HM samples in which the specific HMOS was detected (≥limit of quantification (LOQ)); [2] In only two samples, the respective HMOS was present and hence Q3 could not be calculated; [a,b,c and d] Different superscript letters indicate statistical significance ($p<0.05$). Significance values have been adjusted by the Bonferroni correction for multiple tests.

Abbreviations used: 2′-FL: 2′-fucosyllactose; 3-FL: 3-fucosyllactose; DFL: difucosyllactose; LNT: lacto-N-tetraose; LNnT: lacto-N-neotetraose; LNFP I: lacto-N-fucopentaose I; LNFP II: lacto-N-fucopentaose II; LNFP III: lacto-N-fucopentaose III; LNFP V: lacto-N-fucopentaose V; LNDFH I: lacto-N-

difucohexaose I; LNDFH II: lacto-N-difucohexaose II; 3′-SL: 3-sialyllactose; 6′-SL: 6-sialyllactose; DSLNT: disialyllacto-N-tetraose; LSTa: sialyl-lacto-N-tetraose a; LSTb: sialyl-lacto-N-tetraose b; LSTc: sialyl-lacto-N-tetraose c; 2′-F-LNH: 2′- fucosyllacto-N-hexaose; 3′-F-LNH: 3′-fucosyllacto-N-hexaose

Supplemental Table S4. Percentage of human milk (HM) samples (n=171) in which the specific human milk oligosaccharide (HMOS) was detected (Presence, % of total) and relative peak area (rPA, %) of the selected HMOS (median (Q1-Q3)) according to maternal HM group [4–6]. HM samples were collected longitudinally over the first year postpartum from 24 Dutch mothers.

	**HM Group I (*n* = 126 Samples, 18 Donors)**		**HM Group II (*n* = 21 Samples, 3 Donors)**		**HM Group III (*n* = 15 Samples, 2 Donors)**		**HM Group IV (*n* = 9 Samples, 1 Donor)**	
	**Presence** *% of total*[1]	**rPA (%)** *Median (Q1-Q3)*	**Presence** *% of total*[1]	**rPA (%)** *Median (Q1-Q3)*	**Presence** *% of total*[1]	**rPA (%)** *Median (Q1-Q3)*	**Presence** *% of total*[1]	**rPA (%)** *Median (Q1-Q3)*
2′-FL	100	22.3 (15.0-29.9)^{a}	0	-	100	42.0 (35.5-53.0)^{b}	0	-
3-FL	96	12.2 (6.29-21.7)^{a}	100	35.9 (26.4-45.3)^{b}	33	3.61 (3.04-4.40)^{c}	100	6.78 (3.04-7.31)^{d}
DFL	100	1.86 (1.29-2.50)^{a}	0	-	100	1.01 (0.62-1.48)^{a}	0	-
LNT	100	10.6 (7.87-13.1)^{a}	100	15.3 (11.3-20.6)^{b}	100	9.77 (6.50-13.8)^{a}	100	51.8 (47.8-59.3)^{c}
LNnT	94	1.03 (0.73-1.51)^{a}	71	0.73 (0.55-1.13)^{b}	93	1.22 (0.96-1.80)^{a}	100	1.35 (0.94-1.89)^{a,b}
LNFP I+V	100	7.56 (3.29-12.5)^{a}	100	1.51 (1.36-1.63)	100	18.8 (15.6-24.5)^{b}	100	1.29 (1.16-1.55)
LNFP II	100	5.79 (3.31-9.01)^{a}	100	19.7 (17.1-21.2)^{b}	0	-	0	-
LNFP III	100	7.52 (5.66-9.12)^{a}	100	9.28 (8.06-10.6)^{b}	100	8.94 (6.99-12.3)^{a,b}	100	15.6 (10.1-18.4)^{b}
LNDFH I	100	16.3 (13.5-19.1)^{a}	0	-	0	-	0	-
LNDFH II	6.3	1.20 (1.04-1.85)^{a}	90	3.91 (3.09-4.19)^{b}	0	-	0	-
3′-SL	100	1.08 (0.78-1.43)^{a}	100	1.37 (1.04-1.91)^{a}	100	1.18 (1.04-2.15)^{a}	100	1.76 (1.05-2.21)^{a}
6′-SL	95	0.88 (0.52-1.66)^{a}	95	0.74 (0.51-1.71)^{a}	100	0.91 (0.37-1.62)^{a}	100	1.74 (0.66-2.71)^{a}
DSLNT	100	1.97 (1.40-2.47)^{a}	95	3.30 (2.91-3.75)^{b}	100	2.95 (1.94-4.10)^{a,b}	100	5.53 (5.00-8.83)^{c}
LSTa	46	0.20 (0.17-0.28)^{a}	33	0.26 (0.20-0.31)^{a}	27	0.23 (0.21-0.42)^{a}	100	0.78 (0.75-0.93)^{b}
LSTb	99	0.93 (0.69-1.08)^{a}	100	1.43 (1.29-1.89)^{b}	100	0.64 (0.51-0.98)^{a}	100	2.36 (2.18-2.72)^{c}
LSTc	73	0.57 (0.27-1.08)^{a}	57	0.70 (0.32-1.20)^{a}	60	0.53 (0.29-0.77)^{a}	89	1.17 (0.51-1.64)^{a}
3'-F-LNH	93	2.49 (1.59-3.90)^{a}	86	4.06 (1.23-6.82)^{a,b}	87	2.76 (1.75-4.37)^{a}	100	7.46 (4.94-8.41)^{b}
2'-F-LNH	16	1.00 (0.61-2.14)^{a}	0	-	20	0.90 (0.32- .)[3] ^{a}	0	-
β3′-GL[2]	83	0.087 (0.062-0.15)^{a}	95	0.11 (0.081-0.14)^{a}	73	0.12 (0.11-0.13)^{a}	100	0.42 (0.38-0.51)^{b}
β6′-GL[2]	100	0.36 (0.25-0.53)^{a}	100	0.52 (0.44-0.61)^{b}	100	0.42 (0.38-1.01)^{a,b}	100	1.07 (0.87-1.36)^{c}
α3′-GL[2]	1.6	0.18 (0.13- .)[3] ^{a}	0	-	0	-	0	-

[1] Percentage of HM samples in which the specific HMOS was detected (≥limit of quantification (LOQ)); [2] For β6′-GL, β3′-GL and α3′-GL, the limit of detection was defined at a signal-to-noise ratio (S/N) of 3. For all other HMOS, the limit of quantification was defined at an S/N of 10; [3] In only two samples, the respective HMOS was present and hence Q3 could not be calculated; [a, b, c and d] Different superscript letters indicate statistical significance ($p<0.05$). Significance values have been adjusted by the Bonferroni correction for multiple tests.

Abbreviations used: 2′-FL: 2′-fucosyllactose; 3-FL: 3-fucosyllactose; DFL: difucosyllactose; LNT: lacto-N-tetraose; LNnT: lacto-N-neotetraose; LNFP I: lacto-N-fucopentaose I; LNFP II: lacto-N-fucopentaose II; LNFP III: lacto-N-fucopentaose III; LNFP V: lacto-N-fucopentaose V; LNDFH I: lacto-N-difucohexaose I; LNDFH II: lacto-N-difucohexaose II; 3′-SL: 3-sialyllactose; 6′-SL: 6-sialyllactose; DSLNT: disialyllacto-N-tetraose; LSTa: sialyl-lacto-N-tetraose a; LSTb: sialyl-lacto-N-tetraose b; LSTc: sialyl-lacto-N-tetraose c; 2′-F-LNH: 2′-fucosyllacto-N-hexaose; 3′-F-LNH: 3′- fucosyllacto-N-hexaose; β3′-GL: β3′-galactosyllactose; β6′-GL: β6′-galactosyllactose; α3′-GL: α3′-galactosyllactose.

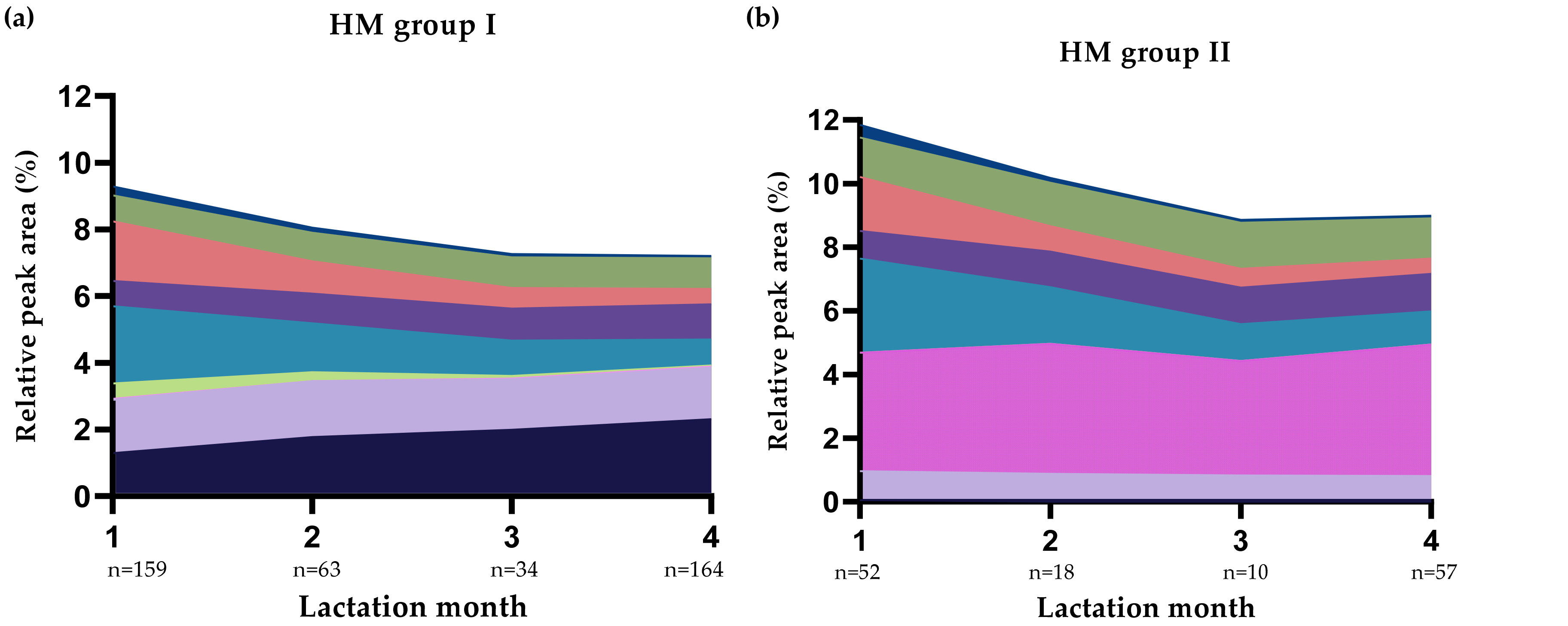

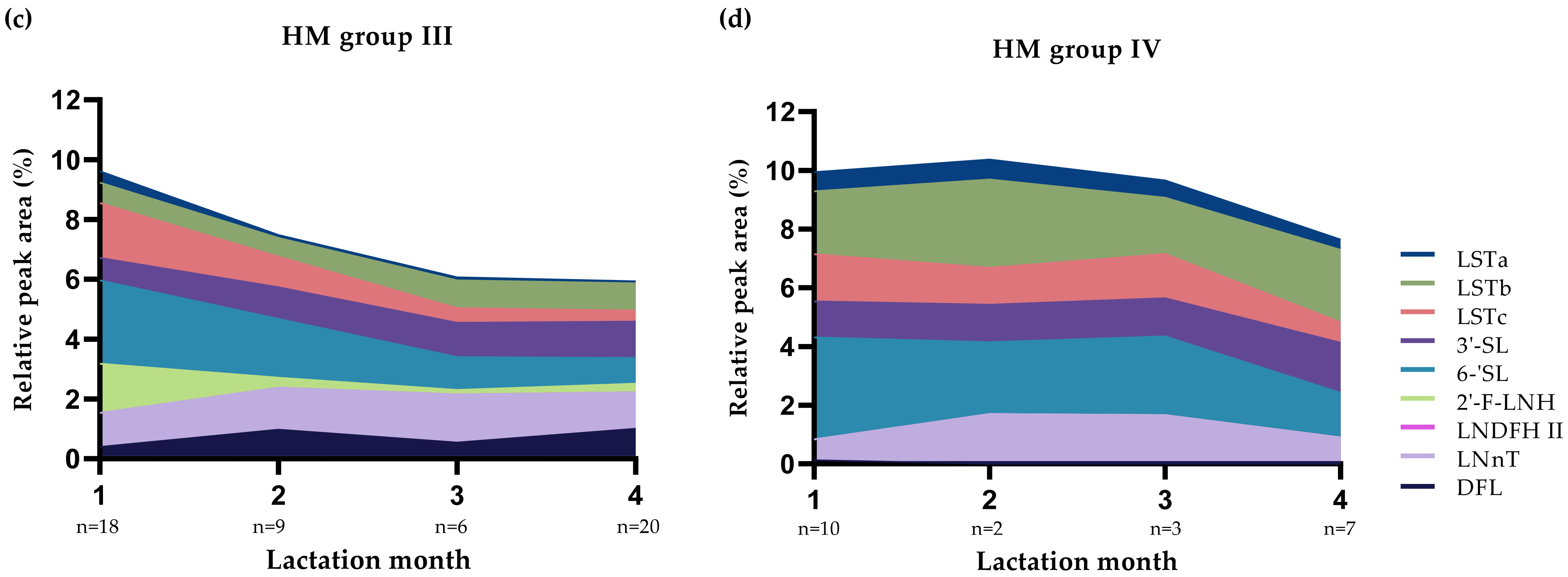

Supplemental Figure S1. Median relative peak areas (rPA) of low-abundant human milk oligosaccharides (HMOS) in the human milk (HM) samples (n=635) collected in the first 4 months of lactation according to HM group [4–6] and lactation stage. (**a**) HM group I; (**b**): HM group II; (**c**): HM group III and (**d**): HM group IV. Three samples could not be assigned to a HM group and are for this reason not depicted in these figures.

References

1. Kottler R, Mank M, Hennig R, Müller-Werner B, Stahl B, Reichl U, et al. Development of a high-throughput glycoanalysis method for the characterization of oligosaccharides in human milk utilizing multiplexed capillary gel electrophoresis with laser-induced fluorescence detection. Electrophoresis. 2013;34(16):2323-36.
2. Melnik LA, Krysenko DA. Ultrapure Water: Properties, Production, and Use. J Water Chem Tech. 2019;41:143-50.
3. Ullsten S, Danielsson R, Backstrom D, Sjoberg P, Bergquist J. Urine profiling using capillary electrophoresis-mass spectrometry and multivariate data analysis. Journal of chromatography A. 2006;1117(1):87-93.
4. Stahl B, Thurl S, Henker J, Siegel M, Finke B, Sawatzki G. Detection of four human milk groups with respect to Lewis-blood-group-dependent oligosaccharides by serologic and chromatographic analysis. Advances in experimental medicine and biology. 2001;501:299-306.
5. Thurl S, Henker J, Siegel M, Tovar K, Sawatzki G. Detection of four human milk groups with respect to Lewis blood group dependent oligosaccharides. Glycoconj J. 1997;14(7):795-9.
6. Thurl S, Munzert M, Henker J, Boehm G, Müller-Werner B, Jelinek J, et al. Variation of human milk oligosaccharides in relation to milk groups and lactational periods. Br J Nutr. 2010;104(9):1261-71.
